# Targeted Disruption of β-Arrestin 2-Mediated Signaling Pathways by Aptamer Chimeras Leads to Inhibition of Leukemic Cell Growth

**DOI:** 10.1371/journal.pone.0093441

**Published:** 2014-04-15

**Authors:** Jonathan W. Kotula, Jinpeng Sun, Margie Li, Elizabeth D. Pratico, Mark P. Fereshteh, Douglas P. Ahrens, Bruce A. Sullenger, Jeffrey J. Kovacs

**Affiliations:** 1 Departments of Surgery and Molecular Genetics and Microbiology, Duke University Medical Center, Durham, North Carolina, United States of America; 2 Departments of Medicine and Biochemistry, Duke University Medical Center, Durham, North Carolina, United States of America; 3 b3 bio, Inc. Research Triangle Park, North Carolina, United States of America; University of Pécs Medical School, Hungary

## Abstract

β-arrestins, ubiquitous cellular scaffolding proteins that act as signaling mediators of numerous critical cellular pathways, are attractive therapeutic targets because they promote tumorigenesis in several tumor models. However, targeting scaffolding proteins with traditional small molecule drugs has been challenging. Inhibition of β-arrestin 2 with a novel aptamer impedes multiple oncogenic signaling pathways simultaneously. Additionally, delivery of the β-arrestin 2-targeting aptamer into leukemia cells through coupling to a recently described cancer cell-specific delivery aptamer, inhibits multiple β-arrestin-mediated signaling pathways known to be required for chronic myelogenous leukemia (CML) disease progression, and impairs tumorigenic growth in CML patient samples. The ability to target scaffolding proteins such as β-arrestin 2 with RNA aptamers may prove beneficial as a therapeutic strategy.

**Highlights:**

- An RNA aptamer inhibits β-arrestin 2 activity.
- Inhibiting β-arrestin 2 impedes multiple tumorigenic pathways simultaneously.
- The therapeutic aptamer is delivered to cancer cells using a cell-specific DNA aptamer.
- Targeting β-arrestin 2 inhibits tumor progression in CML models and patient samples.

## Introduction

β-arrestins are ubiquitously expressed proteins that regulate G protein-coupled receptor (GPCR) or seven transmembrane spanning receptor (7TMR) signaling through receptor desensitization and internalization [1], [2]. β-arrestins have also been shown to be important signaling adaptors and scaffolds that facilitate the activation of numerous effector pathways, such as the mitogen-activated protein kinases and Src [3], [4]. The range of known 7TMR coupled signaling systems which are engaged by β-arrestins has grown rapidly, as has the list of cellular physiological processes which are regulated by these β-arrestin mediated biochemical pathways [1]. Recently, however, an even more surprising development has been the growing list of publications that document roles for the β-arrestins in signaling and/or endocytosis of other families of cellular receptors, and transporters. These include non-receptor and receptor tyrosine kinases, non-classical 7TMRs such as Smoothened [5], [6] and Frizzled [7], [8], and cytokine receptors, such as the TGFβ receptor [9], amongst others [10]. As with the 7TMRs, many of these molecules are shown to interact with the β-arrestins in a ligand- or stimulus-dependent fashion. Moreover, many of these newly discovered interactions are pertinent to, and/or regulate cellular proliferation, differentiation and apoptosis [10]. Unsurprisingly, given these vital roles in numerous signaling mechanisms, β-arrestins have been implicated in a broad range of diseases including asthma [11], idiopathic pulmonary fibrosis [12] and various tumorigenic and metastatic events [13]–[15].

Several exciting and “non-traditional” pathways that involve β-arrestin-mediated signaling have been elucidated over the course of the last decade. One of these signaling cascades is the Hedgehog/Smoothened (Hh/Smo) pathway, in which β-arrestins play a role in facilitating both the endocytosis of Smo from the plasma membrane [5] and the proper intracellular localization of Smo for signaling events [6]. Loss of β-arrestin compromises both signaling and developmental events downstream of the Hh/Smo axis in multiple model systems [10].

Intriguingly, another signaling pathway, the Wingless/Frizzled (Wnt/Fz) signaling axis, also relies on β-arrestin-mediated signaling to promote its physiological effects [10]. In canonical Wnt signaling pathways, β-arrestins interact with Disheveled and Axin, inactivate GSK3β and consequently stabilize β-catenin, thus promoting Wnt/Fz signaling [7], [8]. As with the Hh/Smo signaling axis, loss of β-arrestins leads to an inhibition of intracellular signaling events and physiological responses downstream of Wnt/Fz.

Both the Hh/Smo pathway and the Wnt/Fz pathway have been shown to be required for the onset and maintenance of chronic myelogenous leukemia (CML) [16], [17] suggesting that β-arrestins may play a role in the pathogenesis of hematopoietic malignancies. Indeed, recent work from our group established a crucial role for β-arrestin 2 in the establishment and propagation of the chronic and blast crisis phases of CML [18]. Genetic ablation of β-arrestin 2 prevented both the onset and maintenance of CML and the more advanced blast crisis CML (bcCML). Acute removal of β-arrestin 2 through shRNA-mediated knockdown caused a regression of the diseased phenotype in both animal models and in primary patient samples. This suggests that a therapy targeting β-arrestin 2 in tumor cells which have become addicted to β-arrestin-mediated signaling pathways might prove beneficial to patients with CML. Unfortunately, to date, siRNA or shRNA mediated gene ablation has proven to be ineffective in clinical settings. Additionally, due to the fact that β-arrestin 2 is a ubiquitous scaffolding protein without an enzymatic domain, targeting this protein with a small molecule based inhibitor selectively in diseased cells presents unique challenges requiring a novel approach.

In order to selectively target and inhibit β-arrestin 2, we sought to identify 2′F-RNA aptamers that bind β-arrestin 2. Aptamers are oligonucleotides whose secondary and tertiary structures enable specific and selective binding to large patches on the surface of protein targets and effectively block protein-protein interactions. Thus recently aptamers have emerged as a novel class of viable therapeutics that may be particularly useful in settings where blocking macromolecular assemblies that occur on scaffolding proteins, is expected to impede target protein function [19], [20]. Aptamers have been generated through Systematic Evolution of Ligands by EXponential enrichment, or SELEX, to various protein targets [21], [22]. We performed SELEX to identify aptamers that would bind with high-specificity to β-arrestin 2, and evaluated the ability of these ligands to inhibit the activation of downstream signaling pathways including the Hh/Smo and Wnt/Fz pathways, and thereby prevent leukemic cell growth. However, selectively delivering these β-arrestin 2-targeting aptamers into cancer cells remained a challenge.

Recently, aptamers that recognize cell surface receptors have been utilized to deliver various cargos including toxins, siRNA and splice-switching oligonucleotides into specific cell-types [23]–[28]. Such aptamer-cargo therapies, commonly referred to as aptamer chimeras [29], are ideally suited for oncology settings because they can be engineered to contain two layers of selectivity: (1) an aptamer domain that binds exclusively to a marker over-expressed on cancer cells and (2) a therapeutic agent (toxin, oligonucleotide or aptamer) that affects an essential pathway in tumor but not normal cells.

Along these lines, we have recently utilized a DNA aptamer that specifically delivers oligonucleotides to various cancer cells, including leukemic cells, by binding to the cell surface protein nucleolin and internalizing into the cell [25]. Through this delivery strategy, the DNA ‘targeting aptamer’ directly delivers the RNA ‘therapeutic aptamer’ without any viral or liposomal vector. Interestingly, proteomics studies from our group have found that nucleolin is one of the most common proteins associated with β-arrestin, indicating that the two proteins are in close proximity within cells [30]. This observation presented the unique opportunity to selectively target β-arrestin 2 in cancer cells by linking the nucleolin targeting aptamer to a β-arrestin 2 aptamer (i.e., nucleolin-βarr2 aptamer chimera) through complementary base pair annealing in order to deliver the β-arrestin 2 aptamer into cells.

**Figure 1:**
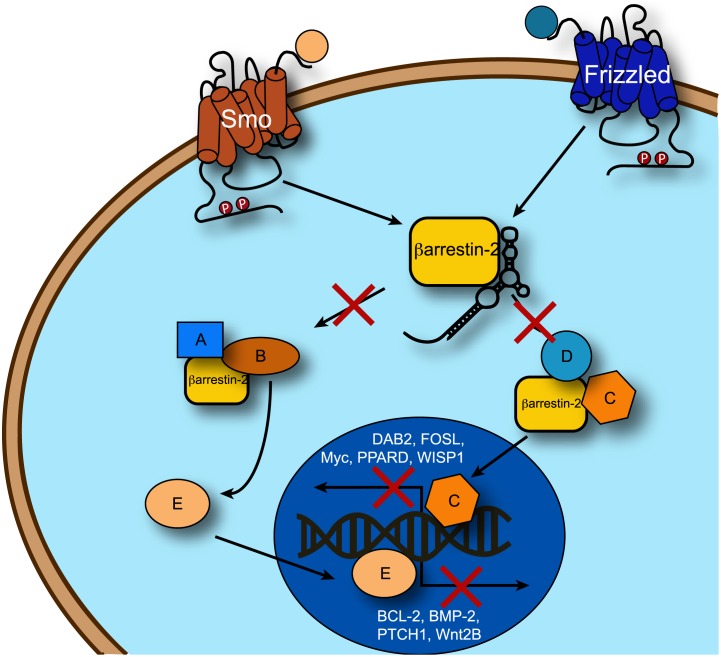
Schematic of signaling pathways inhibited by β-arrestin 2 aptamer binding. The β-arrestin 2 aptamer binds β-arrestin 2 and inhibits its interactions with proteins in both the Hh/Smo and Wnt/Fz signaling pathways. This significantly decreases the transcription of genes controlled by these pathways, preventing diseased cells from replicating. For a detailed review of these pathways see [10].

Based on the properties of the individual components of the nucleolin-β-arr2 chimera and the role of β-arrestin 2 signaling in CML disease progression, we hypothesized that this novel aptamer chimera would selectively inhibit multiple β-arrestin 2-mediated signaling pathways in leukemic cells (Figure 1), thereby blocking their self-renewal capacity. Indeed, as we show here, the nucleolin-βarr2 aptamer chimera was able to deliver βarr2 targeting aptamers into cells, inhibit signaling and decrease the tumorigenic potential of leukemic cells.

## Results

### Development of a Specific β-arrestin 2-binding Aptamer

**Figure 2:**
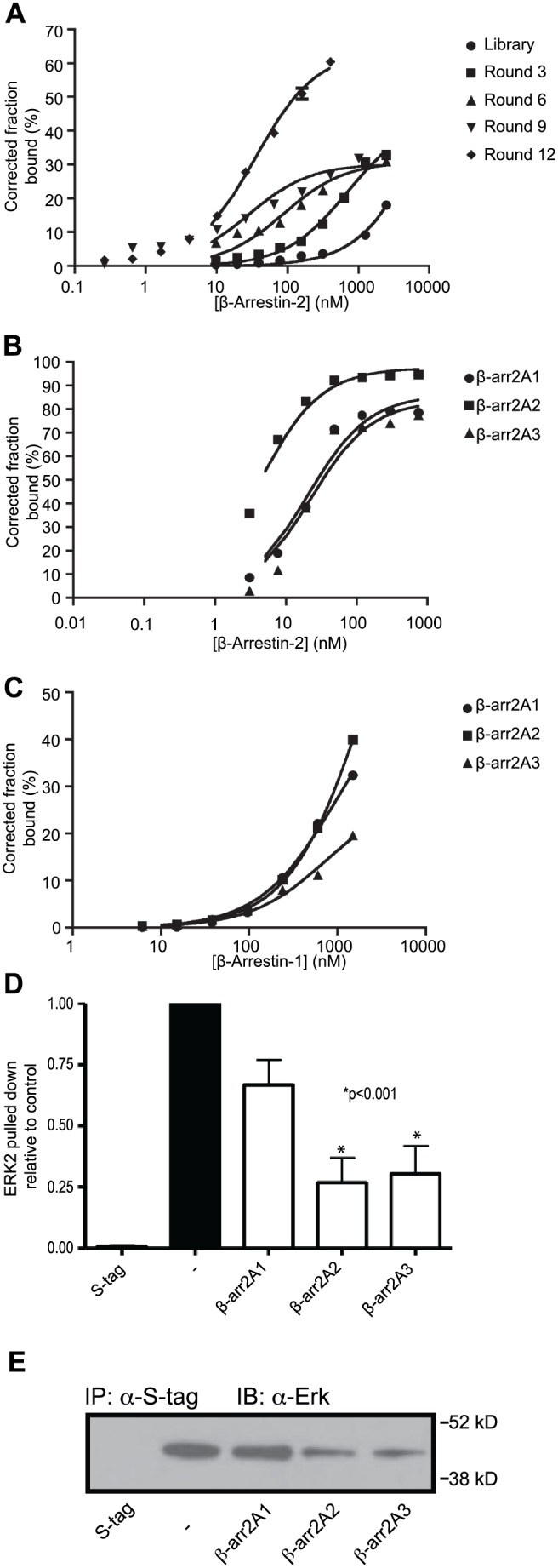
Development of the β-arrestin 2 aptamer. (A) SELEX was performed on a random library of 10^14^ RNA oligonucleotides against β-arrestin 2. Each round, the pool of aptamers that had affinity for β-arrestin 2 was enriched, and the binding affinity of each round as measured by its dissociation constant (Kd) increased. Aptamers from round 9 and 12 were cloned and analyzed. (B) The three aptamers that bound β-arrestin 2 with the highest affinity were β-arr2A1 (Kd = 19.83 nM, Bmax = 86.04), β-arr2A2 (Kd = 4.13 nM, Bmax = 97.38), and β-arr2A3 (Kd = 22.03 nM, Bmax = 83.61). (C) Those aptamers showed selectivity for β-arrestin 2, as they were very poor binders of β-arrestin 1 in comparison, β-arr2A1 (Kd = 965.5 nM, Bmax = 53.99), β-arr2A2 (Kd = 2159.0 nM, Bmax = 97.31), and β-arr2A3 (Kd = 748.5 nM, Bmax = 22.58). (D) *In vitro* interactions between purified β-arrestin 2 and its cytoplasmic binding partner Erk were measured in the presence or absence of β-arrestin 2 binding aptamers and then precipitated with S-tag beads. Coimmunoprecipitation of recombinant Erk was visualized by western blot and then quantified and compared to a control reaction. β-arr2A2, and β-arr2A3 significantly reduced the interaction between β-arrestin 2 and Erk. Representative blot image shown.

Several methods have been developed to interrogate β-arrestin functions in cells. These include loss of β-arrestin 2 function through the use of genetic knockout or RNAi-mediated silencing and the use of biased receptor ligands in order to selectively activate β-arrestin 2-mediated signaling over other pathways [31]. While these methods serve as useful tools for interrogating β-arrestin biology, they do not offer significant promise for therapeutic development for blocking aberrant β-arrestin-mediated signaling. As such, we set out to identify a small RNA aptamer that bound to and inhibited β-arrestin 2 with the intent of finding a molecule that could inhibit β-arrestin 2-mediated signaling if properly delivered to a cell. To this end we performed iterative RNA aptamer selections [21] using a library containing approximately 10^14^ RNA oligonucleotides, in order to identify species capable of binding purified β-arrestin 2 with high affinity *in vitro*. From Round 3 through Round 9 of the selection process, the binding affinity (K_d_) of enriched RNA pools increased gradually with each subsequent round of selection (Figure 2A). Eventually, Round 12 (K_d_ = 35 nM) showed no increase in affinity over Round 9 (K_d_ = 28 nM), suggesting that the selection process had reached a plateau and the aptamer pool was concentrated with high affinity binders for β-arrestin 2.

We subsequently isolated 25 unique sequences from Round 9 and 32 unique sequences from Round 12. The binding affinities for these individual clones were analyzed and three aptamers were identified that bound with high affinity to β-arrestin 2 (hereafter termed β-arr2As), while they bound with low affinity to β-arrestin 1 (Figure 2B and C). Specifically, the binding affinities (K_d_) of aptamers β–arr2A1, β–arr2A2 and β–arr2A3 for β-arrestin 2 were 19.83 nM, 4.13 nM and 22.03 nM, respectively (Figure 2B). By contrast, aptamers β–arr2A1, β–arr2A2 and β–arr2A3 exhibited approximately 35–500 fold lower binding affinities for β-arrestin 1 (K_d_ = 965.5 nM, 2159 nM and 748.5 nM, respectively). These data demonstrate that aptamers β–arr2A1, β–arr2A2 and β–arr2A3 tightly and selectively bind to β-arrestin 2.

One of the most important features of β-arrestin 2, from an intracellular signaling standpoint, is its ability to scaffold and assemble signaling complexes. For example, β-arrestin 2 forms numerous complexes with downstream effectors such as Erk, Src and PDE. We hypothesized that the isolated RNA aptamers that tightly bound β-arrestin 2 might impair its ability to form complexes with its downstream signaling effectors. To test this, we developed *in vitro* co-immunoprecipitation assays in which recombinant S-tag-β-arrestin 2 was incubated with recombinant proteins such as ERK in the absence or presence of β–arr2A aptamers. The co-immunoprecipitation was performed using anti-S-tag antibody and membranes were blotted for the presence of Erk and β-arrestin 2. As shown in Figure 2D, we observed robust β-arrestin 2-Erk complex formation in the absence of aptamer. Interestingly, despite all three aptamers having similar affinity for β-arrestin 2, only β–arr2A2 and β–arr2A3 inhibited formation of the β-arrestin 2-Erk complex when present at 100 nM, a value 5-fold greater than the calculated Kd. However, all three aptamers inhibited *in vitro* complex formation between β-arrestin 2 and PDE (data not shown). Together, these data show that β-arrestin 2 aptamers inhibit β-arrestin 2 signaling complex formation, thereby implying that these small RNA oligonucleotides could potentially disrupt β-arrestin 2-mediated signaling within cells. Moreover, the varying ability of these aptamers to inhibit the formation of different signaling complexes suggests that they may have distinct binding domains on β-arrestin 2 or stabilize different conformations of β-arrestin 2.

### Intracellular Delivery of a β-arrestin 2-targeting Aptamer Chimera

**Figure 3:**
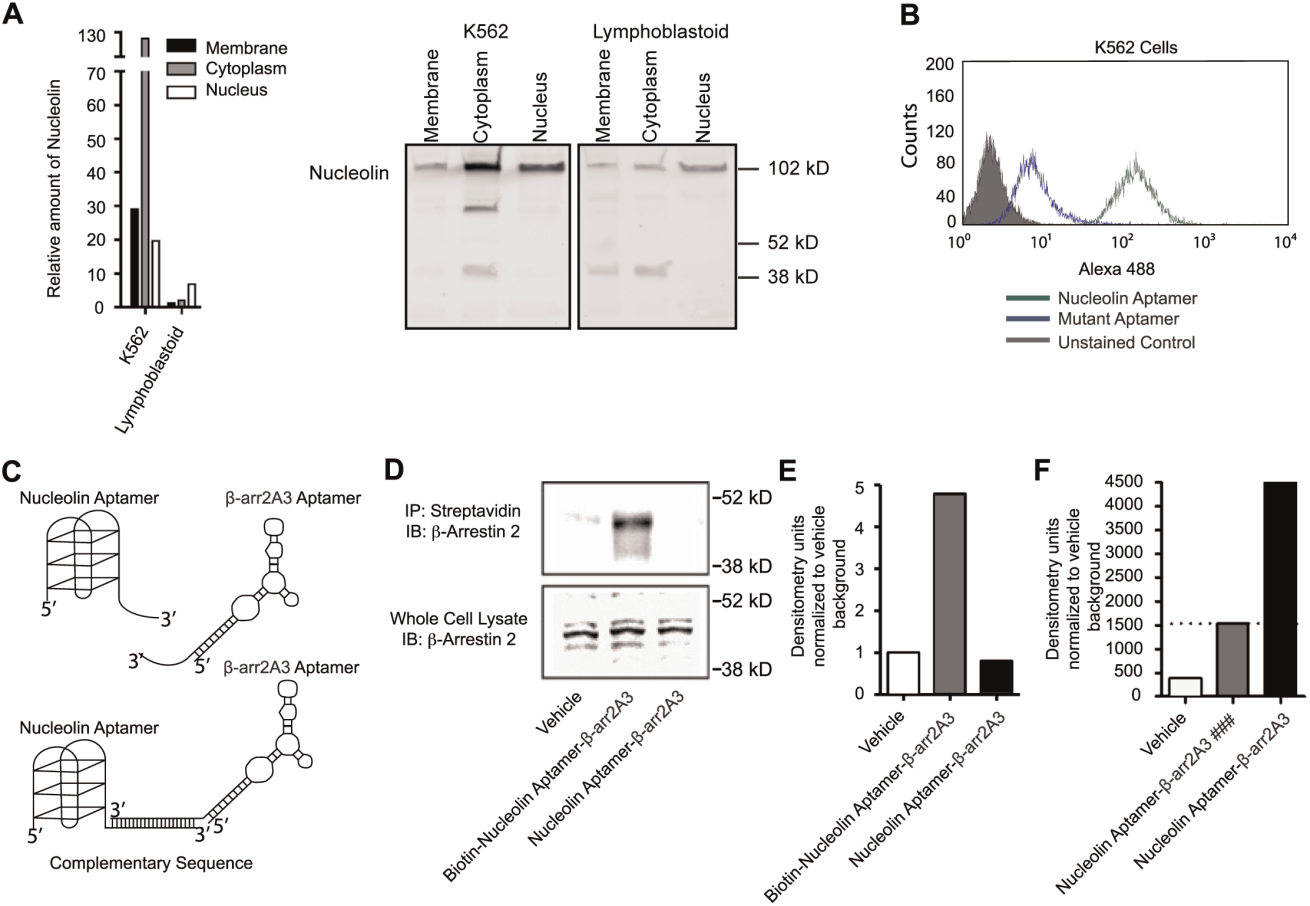
Construction and delivery of an internalizing β-arrestin 2 aptamer chimera. (A) Comparative western analysis was performed on the nucleolin protein levels of different cellular fractions in two different cell types. K562 cells, a CML cell-line, had approximately 30x more membrane-associated nucleolin than lymphoblastoid cells, which are non-cancerous human B cells. Representative blot image shown. (B) The nucleolin aptamer, which requires membrane-associated nucleolin for cell binding and internalization, bound and internalized into K562 cells as analyzed by flow cytometry. (C) Aptamer chimeras were generated with the nucleolin aptamer and the β-arr2A3 aptamer. The nucleolin aptamer acted as a cell-specific delivery agent, and was joined to the β-arr2A3 aptamer by complementary base pair annealing. (D) Biotinylated β-arr2A3 was hybridized to nucleolin aptamer and then allowed to internalize into cells for 24 hours. Cells were then lysed and subjected to biotin pull-downs by way of streptavidin beads. Coimmunoprecipitated β-arrestin 2 was visualized by western blot. (E) Quantification of β-arrestin 2 signals from (D), representative results of 3 independent experiments are shown. (F) Nucleolin-β-arr2 was applied to K562 cells and allowed to internalize for 6 hours. Cells were lysed, and lysates were immunoprecipitated using an anti-β-arrestin 2 antibody. Reactions were then subjected to northern blot analysis and were probed for the presence of the β-arr2A3 aptamer. (### = Control IgG antibody).

We have recently shown that β-arrestin 2 is critical for the onset and maintenance of both the chronic and blast crisis stages of CML (CML and bcCML) in mouse models of theses diseases [18]. Previous work has shown that the nucleolin aptamer can deliver oligonucleotides specifically into cancer cells if nucleolin is present on the membrane of the targeted cells [25]. Accordingly, we analyzed K562 cells, a human Gleevec-resistant bcCML cell-line, and found that membrane-associated nucleolin was approximately 30x more abundant than membrane-associated nucleolin in lymphoblastoid cells, which are non-cancerous human B cells (Figure 3A). We then determined by flow cytometry that the nucleolin aptamer actively bound to the K562 cells (Figure 3B).

In order to selectively deliver the β-arrestin 2 aptamer to cancer cells, we designed a strategy to link them to the nucleolin aptamer through complementary base-pair annealing (Figure 3C). Given the properties of each component of the aptamer chimera, we hypothesized that the nucleolin aptamer would specifically target leukemic cells and deliver the β-arr2As into the cell where they would inhibit β-arrestin 2 function by disrupting β-arrestin 2 signaling complexes.

In order to test the function of our nucleolin aptamer-β-arrestin2 aptamer chimera *in situ*, we took two complimentary, but distinct approaches. In each case we treated K562 cells with 200 nM of nucleolin aptamer-β-arr2A3 chimera. In one case, the 5′ end of the β-arr2A3 aptamer was labeled with biotin through a covalent maleimide-mediated linkage (see materials and methods for a detailed protocol). This biotin-labeled β-arr2A3 aptamer was then hybridized to the nucleolin aptamer and applied to cells, while a non-labeled β-arr2A3 was hybridized to the nucleolin aptamer as a control. The chimeras were allowed to internalize for 24 hours, then cells were lysed and incubated with streptavidin beads. The beads were used to pull down biotin-labeled, internalized β-arr2A3, and subsequently these pull-downs were probed for the presence of β-arrestin 2. As shown in Figure 3, biotinylated nucleolin aptamer-β-arr2A3 co-immunoprecipitates with β-arrestin 2 from cell lysates after internalization (Figure 3D and E). In a second case, using a complimentary, yet distinct approach, unlabeled nucleolin aptamer-β-arr2A3 aptamer chimera co-immunoprecipitates with β-arrestin 2 from cells using a β-arrestin 2 specific antibody [32]. Cell growth was not significantly affected at these time points (Figure S1). When these immunoprecipitations were assessed by northern blot with a probe directed against β-arr2A3, the interaction between β-arrestin 2 and β-arr2A3 was visualized (Figure 3F). Taken together, these data show that the nucleolin aptamer-β-arr2A3 chimera internalizes into cells and delivers the β-arr2A3 aptamer to its intracellular target.

### A β-arrestin 2-targeting Aptamer Chimera Inhibits Cell Signaling Cascades

**Figure 4:**
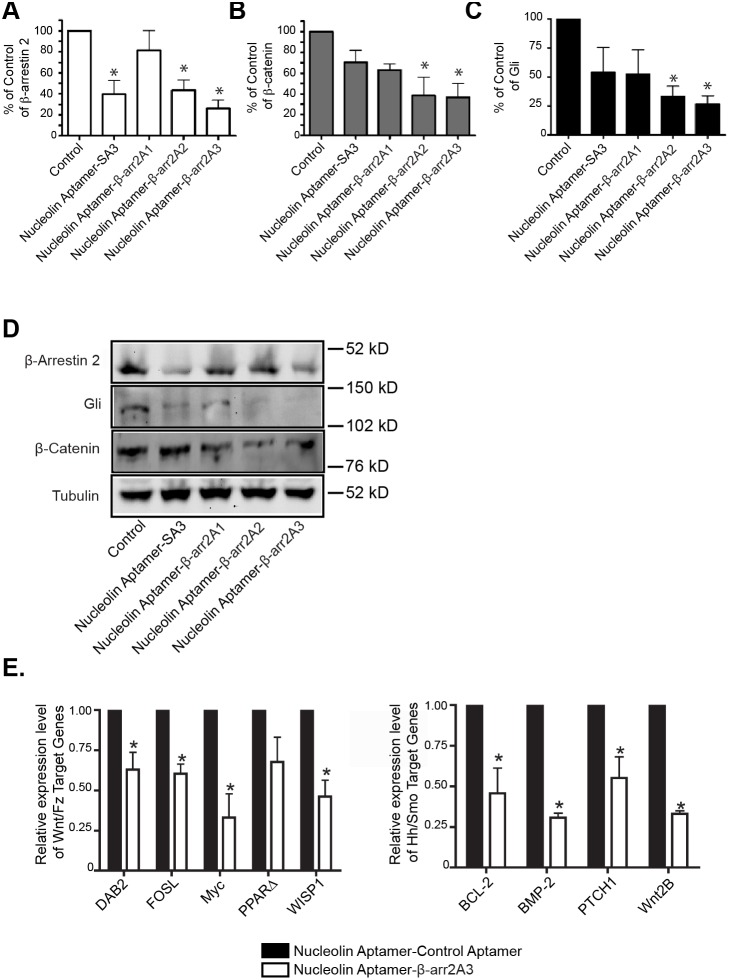
β-arrestin 2 targeting aptamer chimera interrupts multiple signaling pathways in K562 cells. (A–D) K562 cells were treated with 200 nM of indicated aptamer chimeras or vehicle control for 72 hours. Cells were then harvested, lysed and subjected to western blot analysis. β-arrestin 2, Gli, and β-catenin were visualized along with Tubulin as a loading control. n = 5, *p<0.05 using one-way ANOVA with Bonferroni correction. (A) Quantification of β-arrestin 2 protein levels. SA3 = β-arrestin siRNA (B) Quantification of activated Gli protein levels. (C) Quantification of activated β-catenin protein levels. (D) Representative western blot from these experiments. (E) K562 cells were treated with 200 nM of nucleolin-β-arr2A3 or a control chimera and incubated for 72 hours. Total RNA was then purified from these cells and subjected to RT-PCR analysis as described in materials and methods. *Left-panel* – Downstream targets of the Wnt/Fz signaling axis. *Right panel* – Downstream targets of the Hh/Smo signaling axis. *p<0.05 using one-way ANOVA with Bonferroni correction.

In order to test the efficacy of these chimeras for limiting β-arrestin 2 activity in various signaling pathways, we applied the chimera constructs (200 nM) to K562 cells for 72 hours. After application, cells were collected, lysed and subjected to western blotting for β-arrestin 2, the active form of β-catenin (as a marker of Wnt/Fz pathway activity), and the activated form of Gli (as a marker of Hh/Smo pathway activity) (Figure 4). The application of a nucleolin aptamer-β-arrestin 2 siRNA chimera (nucleolin aptamer-SA3) was effective in reducing β-arrestin 2 protein levels (Figure 4A and D). However, it failed to significantly decrease levels of active β-catenin and Gli (Figure 4B–D). Interestingly, a subset of our nucleolin aptamer-β–arr2A chimeras including β-arrA2 and β-arrA3 decreased β-arrestin 2, as well as both β-catenin and Gli levels in K562 cells (Figure 4B–D), suggesting that these aptamers were delivered to and inhibited the activity of β-arrestin 2. Of note, β-arrA1 had little effect (Figure 4A–D) and underscores a difference in the inhibitory properties of these chimeras. Based on the data presented here, we determined that β-arr2A3 was the most potent inhibitor of β-arrestin 2 function in leukemic cells and thus proceeded to interrogate its properties further.

To further validate our hypothesis that β-arr2A3 was specifically inhibiting signaling downstream of both the Hh/Smo and Wnt/Fz pathways, we treated K562 cells with the nucleolin aptamer-β-arr2A3 chimera or a control molecule where the β-arr2A3 aptamer was replaced with a non-specific RNA aptamer (sequence provided in materials and methods). After 72 hours, we examined downstream target genes of these two pathways using an RT-PCR array approach. Downstream of the Wnt/Fz axis, we examined eight target genes. Of those eight, five were down-regulated, and three were unchanged. Of the five genes that were down-regulated, four achieved statistical significance (Figure 4E), including Disabled 2 (DAB2), fos ligand (FOSL), Myc and Wnt-inducible signaling protein 1 (WISP1). Among them, DAB2 and Myc have been implicated in the progression of myelogenous leukemia [33], [34]. Downstream of the Hh/Smo axis, we examined the expression of nine genes. Of these genes, four were significantly down-regulated, while one was unchanged and four were undetectable in either the control-treated or nucleolin aptamer-β-arr2A3-treated samples (Figure 4E). The four genes downstream of the Hh/Smo axis which were down-regulated are B-cell CLL/lymphoma 2 (BCL-2), Bone-Morphogenic Protein-2 (BMP-2), Patched1 (PTCH1) and wingless-type MMTV integration site family, member 2B (Wnt2B). These genes have been shown to play a role in, or serve as biomarkers of leukemic disease [35]–[38]. Importantly, these data confirm that by targeting β-arrestin 2 with an aptamer that impedes the assembly of macromolecular complexes, we are able to inhibit its intracellular signaling function and subsequently reduce the activity of multiple pathways that are important for the onset and progression of CML and bcCML.

### Blockade of β-arrestin 2-dependent Signaling Inhibits Leukemic Cell Growth

**Figure 5:**
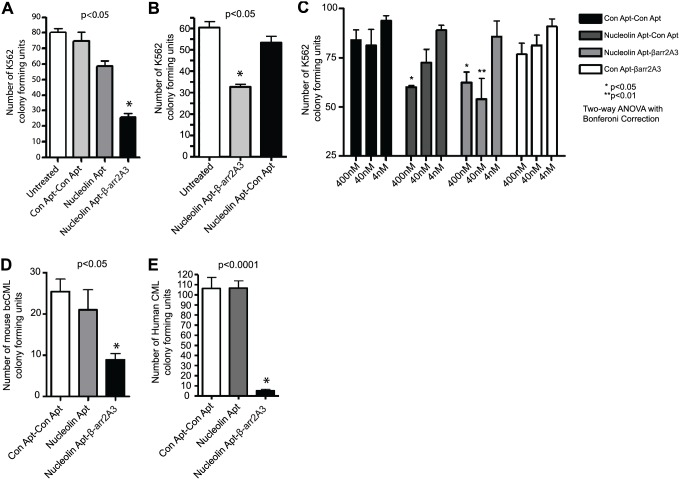
Inhibition of leukemic cell growth via β-arrestin 2 inhibition. (A–B) K562 cells (1000 cells per well) were plated in triplicate in methylcellulose and treated with 40 nM of the indicated aptamer chimera. Cells were incubated for 14 days, and then colonies were counted. n = 5 (C) K562 cells were treated with 400 nM, 40 nM or 4 nM of the indicated aptamer constructs. Cells were plated in duplicate and incubated for 14 days, and colonies were counted. n = 4. (D) Mice were infected with the BCR-ABL transgene and allowed to develop CML as described [18]. Leukemic cells were purified from mouse spleens and treated with the indicated aptamer chimeras at 40 nM. Cells were plated triplicate in methylcellulose and incubated for 14 days before colony forming units were counted. n = 5 animals. (E) Blood samples from human patients were collected and enriched for CD34+ leukemia cells. Cells were treated with the indicated aptamer chimeras at 400 nM and plated in triplicate. After a14 day incubation, colonies were counted. n = 4 patients. All p-values generated using one-way ANOVA with Bonferroni correction.

Armed with the knowledge that our β-arrestin 2 targeting chimera delivered β-arr2A3 to β-arrestin 2 *in situ* and resulted in inhibition of β-arrestin 2 mediated signaling, we set out to examine the ability of the chimera to inhibit leukemic cell growth. We first tested the chimera, and relevant controls, in a colony formation assay, which measures the clonogenic potential of leukemic cells in a semi-solid growth media. Here we used K562 cells, which are a Gleevec-resistant bcCML line. Application of the nucleolin aptamer-β-arr2A3 chimera significantly inhibited the clonogenic potential of K562 cells at a single 40 nM dose; while relevant controls, including the nucleolin aptamer alone, or various aptamer chimera constructs with either a control delivery aptamer replacing the functional nucleolin aptamer, or a control non-βarr2 targeting aptamer replacing the functional β-arr2A3 aptamer, did not significantly inhibit colony formation (Figure 5A–B). It has been reported that the nucleolin aptamer alone can inhibit cell growth at high concentrations through an unknown mechanism [39], so we examined the effect of higher doses of these constructs on colony formation, while still remaining at least 10-fold below doses at which the nucleolin aptamer alone inhibits cell proliferation. As expected, at 400 nM, the nucleolin aptamer carrying a control aptamer inhibited colony formation in the methylcellulose assay (Figure 5C). However, the nucleolin aptamer-β-arr2A3 chimera is at least as effective at 400 nM and more effective at 40 nM (Figure 5A–C) than the nucleolin aptamer alone, thus suggesting that targeted inhibition of β-arrestin 2, through a known mechanism, may prove to be a better target for therapeutic intervention than targeting nucleolin alone.

We also tested whether targeted disruption of β-arrestin 2 would inhibit the growth of primary leukemic samples. To this end, we infected mice as previously described [18] with BCR-ABL to drive CML onset and progression in wild-type animals. Leukemic cells were then harvested from the spleens of these animals after disease onset, and treated once with a control chimera, the nucleolin aptamer alone, or the nucleolin aptamer-β-arr2A3 chimera at 40 nM. Only the β-arrestin 2 targeting chimera was able to inhibit the clonogenicity of these cells (Figure 5D). Even more importantly, this inhibitory effect extended to cells from human leukemia patients. Briefly, CD34+ cells from four CML patients were collected and treated with a single dose of a control chimera, nucleolin aptamer alone, or the nucleolin aptamer-β-arr2A3 chimera and plated in methylcellulose. Again, the nucleolin aptamer-β-arr2A3 chimera robustly attenuates colony formation, thus strongly supporting the idea that targeted disruption of β-arrestin 2-mediated signaling in leukemic cells presents a unique strategy for therapeutic intervention (Figure 5E).

## Discussion

Leukemia is a disease whose onset and progression have been shown to be β-arrestin-dependent events. In fact, the stem cell self renewal events that are characteristic of both CML and bcCML are reliant on multiple β-arrestin-mediated signaling pathways. In a recent publication, we have shown that genetic ablation of β-arrestin 2 in knockout animals protects them from both CML and bcCML in a dramatic fashion [18]. Here, we extend this work to show that targeted disruption of multiple β-arrestin 2 mediated signaling pathways via pharmacologic inhibition blocks critical signaling events in leukemic cells and prevents diseased cells from replicating.

β-arrestins are ubiquitous cellular proteins, which are involved in many signaling pathways. One of the unique features of the β-arrestins that makes it difficult to selectively target one isoform is their high level of homology. In addition, it has been difficult to generate small molecules that antagonize scaffolding proteins. In order to create a tool for examining the potential of pharmacologic inhibition of β-arrestin-mediated signaling, we searched for an RNA aptamer that selectively bound to β-arrestin 2, while showing a low affinity for β-arrestin 1 (Figure 2). A set of aptamers that fit these criteria was isolated and tested for their ability to impede assembly of macromolecular complexes between β-arrestin 2 and downstream signaling factors and subsequent signaling events both *in vitro* (Figure 2) and *in vivo* (Figure 3). Once delivered into leukemic cells using a nucleolin aptamer, we found that the most potent β-arrestin 2 targeting aptamer, β-arr2A3, was able to block multiple signaling pathways concurrently (Figure 4) and inhibit growth of cancerous cells from mice and patients (Figure 5).

The data presented here strongly support the idea that targeting intracellular, non-enzymatic targets is not only tractable, but also feasible using bivalent aptamer containing one aptamer as delivery agent and a second aptamer as direct protein antagonist. Our previous work has shown that the nucleolin aptamer possesses the properties necessary for *in vitro* delivery of oligonucleotide therapeutics to the nuclei of cancer cells [25], and the work herein demonstrates for the first time that nucleolin aptamer chimeras can directly deliver aptamer-based therapeutics without a viral or liposomal vector to intracellular targets in order to generate a therapeutically relevant effect in leukemic cells. Furthermore, many aptamer-derived therapeutic agents are in various stages of clinical trial [40]. Structured oligonucleotides may activate the innate immune response through toll-like receptors (TLRs). However, the nucleolin aptamer’s mechanism of internalization avoids the endosome, which is where oligonucleotide-sensing TLRs are located. Therefore, we believe that this approach will avoid some of the toxicity concerns associated with oligonucleotide therapeutics. Consequently, we believe that by appending other aptamers selected against intracellular targets, the nucleolin aptamer may enhance the utility of an entire class of aptamers that target intracellular proteins.

In addition to the intracellular delivery of payloads by the nucleolin aptamer, the data presented here show that aptamers can affect “undruggable” targets through inhibition of protein-protein interactions. Due to the fact that β-arrestins are non-enzymatic, intracellular, scaffolding proteins that rely on protein-protein interactions to facilitate numerous signaling pathways, traditional drugs such as small molecules, antibodies or synthetic peptides would be expected to be challenging to identify and have limited efficacy. Small molecules are useful for inhibiting enzymatic targets, but are not efficient at inhibiting protein-protein interactions. Antibodies are effective at inhibiting the function of proteins with extracellular domains, but cannot pass through the cell membrane to reach intracellular targets. Short synthetic peptides are able to block protein-protein interactions, but like antibodies, have difficulty crossing the cell membrane to access intracellular targets. Here, we have shown that an aptamer specifically targeting β-arrestin2 can not only inhibit its ability to bind signaling partners, but can be delivered *in situ* where it binds to intracellular β-arrestin2, subsequently inhibiting β-arrestin2-mediated signaling. Although we have not determined the long-term stability of the aptamers within the target cells, β-arrestin2 was inhibited for a period long enough suppress the self-renewal/tumorigenic capacity of leukemic cells. Due to the fact that this process requires the expression of differentiation factors, it would seem that these aptamers have a prolonged existence within cells. These results indicate that aptamers can impact intracellular targets involved in macromolecular assemblies that have been difficult to antagonize through traditional methods.

Aptamers have been selected against intracellular proteins, but have had little use as therapeutic agents, in part because previous to this work, they could not be delivered to specific cells or with high efficiency. Additionally, aptamer processing during systemic administration of aptamer therapeutics remains a problem that is being investigated. However, targeted delivery of aptamers against intracellular targets to aberrant disease causing cells could be achieved through hybridization to other aptamers (as shown here) or to other delivery agents such as monoclonal antibodies. Various formulations of these aptamer containing therapeutic agents could mitigate stability, clearance and toxicity issues. Through a multi-step process, aptamers may be generated to a target, linked to a cell-specific delivery agent, and delivered to an intracellular target to inhibit protein-protein interactions that are critical for disease onset and maintenance, as is shown here to be the case with β-arrestin 2.

While it may seem that the involvement of β-arrestin 2 in a myriad of signaling pathways may act as a roadblock to therapeutic targeting strategies, the data presented here show the power of identifying and targeting diseased cell types that have come to rely on multiple β-arrestin-signaling pathways. Consequently, these cells may be uniquely sensitive to the loss of β-arrestin 2 function, and thus offer new avenues of therapeutic intervention in the future. More generally, this strategy may be amenable to other targets that are common to multiple tumorigenic pathways in diseased cells and may allow for the down-regulation of these pathways with a single therapeutic agent rather than a panel of agents targeting individual signaling cascades.

## Methods and Materials

### Clinical Materials

Our protocol to use clinical patient samples was approved by the DUHS IRB. Samples used in this manuscript were procured with the appropriate informed consent forms and their approval has been attached to this submission.

### Oligonucleotide Sequences

β-arr2A1: 5′- GGG AGG ACG AUG CGG AUU CCG UUA AGU AUC GCG UUA AAC GCU AUG CGG ACG CGU CAG ACG ACU CGC UGA GGA UCC GAG A -3′.

β-arr2A2: 5′- GGG AGG ACG AUG CGG CCA GGU GUA GAC AGA CGU GAG AGA UUG ACC UGG CAG CAG CCA GAC GAC UCG CUG AGG AUC CGA GA -3′.

β-arr2A3: 5′- GGG AGG ACG AUG CGG AUC CUC GUC CCG UCA CGG CAG AAC CAC GUC AGG CCU UCA ACA GAC GAC UCG CUG AGG AUC CGA GA -3′.

Nucleolin Aptamer: 5′- TGG TGG TGG TGG TTG TGG TGG TGG TGG -3′.

SA3: 5′ - CCA ACC UCA UUG AAU UUG AdTdT – 3′.

Control siRNA: 5′- UCA AGA AGC CAA GGA UAA U -3′.

Control Aptamer: 5′- GCC UGG CCU CCU GCA GUG CCA CGC UAU AUU AUC CUA GCG UUC UUG UGU UGG ACA CUC CUA GUG GUA GAG UUC AAG AAC GCA AGG AUA AUU -3′.

Mutant Aptamer: 5′- TCC TCC TCC TCC TTC TCC TCC TCC TCC -3′.

DNA, and DNA-RNA hybrid aptamers were ordered from IDT (Integrated DNA Technologies, Coralville, IA 52241). RNA aptamers were transcribed by *in vitro* transcription using 2′F modified pyrimidines (Trilink Biotechnologies, San Diego, CA), and T7 (Y693F) polymerase.

### Selex

The sequence of the starting RNA combinatorial library was 5′- GGGAGGACGATGCGG-N40-CAGACGACTCGCTGAGGATCCGAGA-3′, where N40 represents 40 random nucleotides. 2′F cytidine triphosphate and 2′F uridine triphosphate (Trilink Biotechnologies, San Diego, CA) were incorporated into the RNA libraries by in vitro transcription in order to confer nuclease resistance. The selection was carried out in selection buffer F (20 mM HEPES, pH 7.4, 150 mM NaCl, 1 mM CaCl_2_, 1 mM MgCl_2_ and 0.01% bovine serum albumin (BSA) at 37°C until round 12. RNA- β-arrestin 2 complexes were separated from unbound RNA by passing them through a nitrocellulose filter (BA 85, Whatman Inc., Florham Park, NJ). Twelve rounds of SELEX were performed on the purified protein. A counter-selection against β-arrestin 1 was performed to remove aptamers that bound β-arrestin 1 and enrich for aptamers that specifically bound β-arrestin 2.

### Cell Culture and Primary Cell Isolation

K562 cells were purchased from ATCC and cultured in 10% FBS IMDM. Studies on primary human bcCML samples were carried out with approval from the Duke University Institutional Review Board. Mononuclear cells were isolated from peripheral blood samples using density-gradient centrifugation. Our protocol to use clinical samples, was approved by the Duke University Hospital System Institutional Review Board for Clinical Investigations.

### Colony Forming Assays

For all assays, cells were counted and resuspended in sterile PBS pH 7.4, such that 100 µL of the PBS/cell mixture was needed for each well of the colony formation assay. Aptamers were added to the PBS/cell mixture, and methylcellulose was added to this mixture to give the final concentration of aptamer shown in figures. 500 µl of methylcellulose was used per well, and wells were plated in triplicate. For assays using K562 cells, 1000 cells per well were plated in Complete methylcellulose media from Stem Cell Technologies (H4344). Human primary patient samples were enriched for CD34+ by FACS analysis from primary human CML patients, infected with control or β-arr2 lentiviral shRNA, and plated into a 24-well plate (50,000 cells per well) with complete methylcellulose medium (Stem Cell Technologies). For the mouse CML colony formation assays, BCR-ABL+ KLS cells were sorted and plated with complete methylcellulose medium (M3434; Stem Cell Technologies).

### Western Blotting

Cells were grown to near confluence, collected, and fractionated into subcellular components (Subcellular Protein Fractionation Kit for Cells, ThermoScientific, Rockford, IL, 61101). The subcellular fractions were run on a precast polyacrylamide gel (BioRad, Hercules, CA, 94547), along with Full-Range Molecular Weight Rainbow Markers (GE Healthcare Life Sciences, Piscataway, NJ, 08855) then transferred to a PVDF membrane. Western analysis was performed on the membrane with a rabbit polyclonal antibody against human nucleolin (Abcam, Cambridge, MA, 02139), and ECL Plex goat anti-rabbit IgG Cy5 secondary antibody (GE Healthcare Lifesciences, Piscataway, NJ, 08855). The blot was analyzed on a Typhoon 9410 variable mode imager (GE Healthcare Lifesciences, Piscataway, NJ, 08855) and quantified.

### S-tag-pulldowns

S-tag-βarrestin 2 was purified as previously described [41] and incubated with S-tag beads in binding buffer (20 mM Tris-HCL, pH 8.0, 150 mM NaCl, 2 mM DTT, 2 mM EDTA, 2 mM EGTA, 1 mM PNSF, and 0.2 mg/ml benzamindine). After a pre-incubation, 2 µg of purified Erk was added in the presence or absence of 150 nM of the indicated aptamer. The reactions were rocked at 4°C overnight, spun at 1000 rpm, and the supernatant was removed. Beads were washed in 500 µL of reaction buffer 3 times and the subjected to SDS-PAGE analysis. Blots were probed with anti-total Erk antibody.

### Biotin Pulldowns

Biotin was conjugated to the 5′ end of βarr2A3 through a sequence of reactions. First, aptamer was treated with alkaline phosphatase (NEB - M0290L) in a reaction containing 1 µl of buffer, 1 µl of alkaline phosphatase, 0.6 nmol of aptamer and dH_2_O to 10 µl which was incubated for 1 hour at 37°. After incubation, the aptamer was subjected to a kinase reaction using T4 Polynucleotide Kinase (3′ phosphatase minus) (NEB - M0236L) in a reaction containing the entirety of the AlkPhos reaction, 1 µl ATP-g-S, 1 µl of buffer, 2 µl of T4PNK, and 6 µl of dH_2_O, which was incubated at 37°C for 2 hours. 3.25 µl of biotin Maleimide (250 mM final) was then added to the reaction and incubated at 65°C for 2 hours. RNA was EtOH precipitated, dried and dissolved in TE. Biotinylated-βarr2A3 was added to K562 cells (5×10^6^ cells in 500 µl of 10% FBS IMDM) at a final concentration of 200 nM and allowed to internalize for 24 hours. Cells were lysed as previously described in glycerol lysis buffer [6], and lysates were added to 25 µl of streptavidin beads and rocked for 3 hours at 4°C. After rocking, pellets were collected, supernatant removed, and beads were subjected to wash 3 times with lysis buffer. Samples were subjected to SDS-PAGE analysis and probed with anti-βarrestin 2 antibody (A2CT).

### Antibodies

Gli – Rabbit Polyclonal from Rockland Immunochemicals (100–401–223).

β-catenin – Rabbit polyclonal from Abcam (ab6301).

β-arrestin 2– A2CT [32].

Erk (p42/p44) – Rabbit monocolonal from Cell Signaling (mAb#4695).

### Flow Cytometry

Cells in a 12-well plate were incubated with nucleolin aptamer or mutant aptamer at 100 nM for 3 h (37°C, 5% CO_2_) then treated with DNase for 10 minutes to degrade any non-internalized aptamer. Cells were washed with PBS and trypsinized with 0.05% Trypsin for FACs analysis (Becton Dickinson FACSCalibur flow cytometer). The aptamers were conjugated at the 5′ end with Alexafluor 488 (Integrated DNA Technologies, Coralville, IA 52241).

### qRT-PCR

RNA was isolated from K562 cells using the Qiagen RNeasy kit with added DNAse purification according to manufacturer’s adapted protocol using the QiaCube purification rotor. Reverse transcription was performed using the RT^2^ First Strand cDNA Synthesis kit (SABiosciences), and 84 genes were assessed by RT-PCR using the Human Signal Pathway Finder array (RT^2^ Profiler PCR Array PAHS-014A; SABiosciences) according to manufacturers instructions using a MyIQ qRT-PCR machine (BioRad). For analysis, the expression level for each gene of interest (GOI) was calculated as 2^−Ct^ followed by normalization to Hprt1, the housekeeping gene (HKG), using the formula 2^−(Ct GOI - Ct HKG)^. Ultimately the fold change in normalized gene expression was calculated by comparing values from Nuc-barr2A3 treated cells (200 nM) (EXP) to cells treated with a control aptamer chimera construct (CTL) according to the following formula: 2^−ΔCt EXP^/2^−ΔCt CTL^. Values were calculated for replicates of three independent experiments and p-values calculated using one-way ANOVA analysis with Bonferroni correction.

### Northern Blot

Aptamer chimeras were prepared and added to K562 cells at 200 nM. After the time noted, the cells were collected and protein pull-downs were performed. The precipitated proteins were run on a polyacrylamide gel and transferred to a nylon membrane, and UV-fixed. The membrane was incubated with a P-32 radiolabeled probe against β-arrestin 2 aptamer (5′-GAG GAT CCG CAT CGT CCT −3′) then quantified on a Molecular Dynamics Storm 840 Phosphoimager (GE Healthcare Lifesciences, NJ).

## Supporting Information

Figure S1 **K562 cells were seeded 1000 cells per well in a 6-well plate.** Cells were immediately treated with 400 nM of the indicated apatamer. After 96 hours of treatment, cell suspensions were counted using a hemocytometer and values were plotted from three independent experiments. Cells did not undergo a general toxicity or retardation of growth rate in these experiments at the time points shown.
(DOC)
