# Supplementary material for: Targeted Disruption of β-Arrestin 2-Mediated Signaling Pathways by Aptamer Chimeras Leads to Inhibition of Leukemic Cell Growth
Source: PLoS One. 2014 Apr 15;9(4):e93441. doi: 10.1371/journal.pone.0093441 (PMC3988186; doi:10.1371/journal.pone.0093441)
Supplement: Figure S1 — K562 cells were seeded 1000 cells per well in a 6-well plate. Cells were immediately treated with 400 nM of the indicated apatamer. After 96 hours of treatment, cell suspensions were counted using a hemocytometer and values were plotted from three independent experiments. Cells did not undergo a general toxicity or retardation of growth rate in these experiments at the time points shown. (DOC) [file pone.0093441.s001.doc]

**Figure S1** – K562 cells were seeded 1000 cells per well in a 6-well plate. Cells were immediately treated with 400 nM of the indicated apatamer. After 96 hours of treatment, cell suspensions were counted using a hemocytometer and values were plotted from three independent experiments. Cells did not undergo a general toxicity or retardation of growth rate in these experiments at the time points shown.
